# Diseases associated with calcium-sensing receptor

**DOI:** 10.1186/s13023-017-0570-z

**Published:** 2017-01-25

**Authors:** C. Vahe, K. Benomar, S. Espiard, L. Coppin, A. Jannin, M. F. Odou, M. C. Vantyghem

**Affiliations:** 1 Service d’Endocrinologie et Métabolisme, Hôpital C Huriez Centre Hospitalo-universitaire de Lille, 1 rue Polonovski, 59 037 Lille Cedex, France; 2 Service de Biochimie et Biologie Moléculaire, Centre de Biologie-Pathologie, Centre Hospitalo-universitaire de Lille, 1 rue Polonovski, 59 037 Lille Cedex, France; 3 Equipe INSERM 1190 Prise en charge translationnelle du diabète, Lille Cedex, France; 4 Institut EGID (European Genomic Institute for Diabetes), Lille Cedex, France

**Keywords:** *CASR*, Hyperparathyroidism, Hypercalcaemia, Hypocalcemia, Hypercalciuria, Hypocalciuria, Hypoparathyroidism, Calcimimetics, Calcilytics

## Abstract

The calcium-sensing receptor (CaSR) plays a pivotal role in systemic calcium metabolism by regulating parathyroid hormone secretion and urinary calcium excretion. The diseases caused by an abnormality of the CaSR are genetically determined or are more rarely acquired. The genetic diseases consist of hyper- or hypocalcemia disorders. Hypercalcaemia disorders are related to inactivating mutations of the *CASR* gene either heterozygous (autosomal dominant familial benign hypercalcaemia, still named hypocalciuric hypercalcaemia syndrome type 1) or homozygous (severe neonatal hyperparathyroidism). The A986S, R990G and Q1011E variants of the *CASR* gene are associated with higher serum calcium levels than in the general population, hypercalciuria being also associated with the R990G variant. The differential diagnosis consists in the hypocalciuric hypercalcaemia syndrome, types 2 (involving *GNA11* gene) and 3 (involving *AP2S1* gene); hyperparathyroidism; abnormalities of vitamin D metabolism, involving *CYP24A1* and *SLC34A1* genes; and reduced GFR. Hypocalcemia disorders, which are more rare, are related to heterozygous activating mutations of the *CASR* gene (type 1), consisting of autosomal dominant hypocalcemia disorders, sometimes with a presentation of pseudo-Bartter’s syndrome. The differential diagnosis consists of the hypercalciuric hypocalcaemia syndrome type 2, involving *GNA11* gene and other hypoparathyroidism aetiologies. The acquired diseases are related to the presence of anti-CaSR antibodies, which can cause hyper- or especially hypocalcemia disorders (for instance in APECED syndromes), determined by their functionality. Finally, the role of CaSR in digestive, respiratory, cardiovascular and neoplastic diseases is gradually coming to light, providing new therapeutic possibilities. Two types of CaSR modulators are known: CaSR agonists (or activators, still named calcimimetics) and calcilytic antagonists (or inhibitors of the CasR). CaSR agonists, such as cinacalcet, are indicated in secondary and primary hyperparathyroidism. Calcilytics have no efficacy in osteoporosis, but could be useful in the treatment of hypercalciuric hypocalcaemia syndromes.

## Background

The calcium-sensing receptor (CaSR), a G-protein coupled receptor (GPCR) family member, is ubiquitously expressed, but mostly in the parathyroid gland and the renal tubule. It enables CaSR-expressing cells to sense alterations in the level of blood calcium and to normalize its concentration, by regulating parathyroid hormone (PTH) secretion and urinary calcium excretion. The CaSR is able to bind numerous ligands, to interact with multiple G-proteins, and to regulate highly divergent downstream signalling pathways and cell fate, through epigenetics and miRNA [1]. Besides Ca^2+^, the ligands include other divalent cations such as Mg^2+^, Ba^2+^, Mn^2+^, Ni^2+^, Sr^2+^ and trivalent cations La^3+^ and Gd^3+^, basic peptides (such as poly-arginine, protamine, and poly-lysine), glutathione and its γ-glutamyl peptides, agonists (such as AMG 416), antagonists and drugs [2]. The human *CASR* gene localizes on chromosome 3q and has 8 exons, the first (1A and 1B) encoding alternative 5′-untranslatesd regions splicing. The CASR promoters are responsive to 1,25-dihydroxyvitamin D, proinflammatory cytokines (TNF-alpha, IL-1beta and IL-6) and the transcription factor glial cells missing-2 (GCM2) [1]. Abnormal CaSR function affects the development of both calciotropic disorders, and non-calciotropic disorders, such as cardiovascular disease and cancer [3].

Several disorders of calcium sensing arise from inherited or acquired abnormalities that ‘reset’ the serum calcium concentration upwards or downwards. They are expressed through a hyper- or hypocalcaemic syndrome [3] (Table 1).

**Table 1:** Main diseases related to CaSR anomalies

Hypocalciuric hypercalcaemia syndromes:	
- Genetic via inactivating mutations of the *CASR* gene o heterozygous (familial benign hypercalcaemia), NB: Serum calcium levels of the variants A986S, R990G and Q1011E slightly higher than in the general population o homozygous, compound heterozygous (severe neonatal hyperparathyroidism) - Acquired via anti-CaSR blocking antibodies (rare)	
Hypercalciuric hypocalcaemia syndrome, more rare, - Genetic via heterozygous activating mutations of the *CASR* gene o autosomal dominant o sometimes with presentation of pseudo-Bartter’s syndrome - Acquired via anti-CaSR stimulating antibodies	
Other disorders - Hypercalciuria - lithiases R990G variant of CaSR - Cancers: tumor suppressor or oncogenic (colon, breast, prostate, neuroblastoma, etc.) - Metabolic syndrome - Hypergastrinaemia - Inflammatory digestive and respiratory diseases - Taste (kokumi)	

Familial hypocalciuric hypercalcaemia syndromes are schematically related to inactivating mutations of the *CASR* gene:

- heterozygous (benign familial hypercalcaemia)
- homozygous (neonatal hyperparathyroidism)

Hypocalcaemia, which is more rare, is related to heterozygous activating mutations of *CASR*, corresponding to autosomal dominant hypocalcaemia, sometimes with a presentation of pseudo-Bartter’s syndrome. Acquired diseases, which are much more rare, are associated with the presence of CaSR-stimulating or CaSR-blocking antibodies.

Finally, the role of CaSR in many diseases that do not cause calcium and phosphate disorders is gradually coming to light, thus providing new therapeutic possibilities.

The aim of this review is 1) to make a point on the different disorders of calcium metabolism associated with CaSR anomalies, their main differential diagnoses, and their treatment, 2) to unveil the less known fields in which the CaSR could be involved. To do so, we performed a literature review with the keywords calcium-sensing receptor, hypocalciuric hypercalcaemia, hypocalcemia and hyperparathyroidism.

## Genetic causes

### Hypercalcaemia via inactivating *CASR* mutations

#### Heterozygous inactivating mutations

##### Typical clinical and laboratory presentation

Heterozygous inactivating *CASR* mutations result in a familial hypocalciuric hypercalcaemia syndrome (FHH) consisting of:

- moderate hypercalcaemia, usually below 1.25 mmol/L,
- a relative hypocalciuria with calcium to creatinine clearance ratio below 0.01, with a gray zone between 0.011 and 0.019 when the diagnosis is still possible [4, 5],
- a normal or high plasma PTH value,
- the usual absence of complications related to this hypercalcaemia.

Familial hypocalciuric hypercalcaemia syndrome, also known as FHH1, familial benign hypercalcaemia or Marx-Auerbach syndrome, is usually transmitted as an autosomal dominant trait. The hypercalcaemia is moderate and asymptomatic, although it may be marked and/or clinically evident in 10% of cases. It persists throughout the patient’s life. Plasma PTH is normal in 80% of cases and is therefore maladjusted to the serum calcium levels. Moderate hypermagnesemia is often seen. A family history of hypercalcaemia should be investigated through the plasma assay of serum calcium in relatives.

##### Clinical forms

Although this form of hypercalcaemia is usually asymptomatic, cases of pancreatitis and chondrocalcinosis have been reported in some adults [6].

An increase in parathyroid volume is generally not seen. However, a dozen cases of parathyroid adenomas associated with *CASR* mutations have been reported, with two families having adenoma and/or familial hyperplasia of the parathyroids with papillary microcarcinoma [7–14]. Moreover, the R990G variant of CaSR seems to be more common in the general Chinese population, but also in Chinese patients with hyperparathyroidism [15]. The coexistence of hyperparathyroidism and hypocalciuric hypercalcaemia syndrome was also identified in 4 out of 139 patients from a hyperparathyroid Caucasian population [16]. The serum calcium levels decreased postoperatively. These associations remain rare and may be found fortuitously. Nevertheless, mutant sensors in the plasma membrane have been shown to be major contributors to hyperplasia of parathyroid glands [17].

Also, a tendency for hypercalciuria has been reported with the R990G variant, also resulting in slightly higher serum calcium levels than in the general population [18–20].

Finally, some cases of recessive inheritance have been reported [21, 22].

##### Differential diagnosis

The differential diagnosis consists of the hypocalciuric hypercalcaemia syndrome types 2 and 3; hyperparathyroidism, familial in particular; abnormalities of vitamin D metabolism; and reduced GFR (glomerular filtration rate) (Table 2).

**Table 2:** Main differential diagnoses of calcium disorders related to *CASR* gene mutations

**Hypercalcaemia**	
Familial hypocalciuric hypercalcaemia (FHH) syndromes	
- type 2 via mutations of the *GNA11* gene (10%) - type 3 via mutations of the *AP2S1* gene (15%) - via mutations of other as of yet unknown genes (20%)	
Familial hyperparathyroidism	
- via mutations of tumour suppressor genes: o *MEN1* (Multiple endocrine neoplasia type 1) o *HRPT2 (/CDC73)* o *CDKN1B* (MEN4) o but also the *APC, SFRPs, GSK3β, RASSF1A, HIC1, RIZ1,* and *WT1* genes, and possibly *CASR*, *GNA11*, *AP2S1, and GCM2*… - via mutations of proto-oncogenes (*CCND1/PRAD1, RET* [MEN2]*, ZFX, CTNNB1, EZH2)cpe*	
Hyperparathyroidism with normal or low PTH with hypercalciuria via mutation of the genes	
- *CYP24A1*	
- *SLC34A1*	
Hypercalcemia associated with reduced glomerular filtration rate, making the low urine calcium difficult to interpret	
**Hypocalcaemia**	
Hypercalciuric hypocalcaemia syndrome type 2 via activating mutation of the *GNA11* gene.	
No gain-of-function mutation described for the *AP2S1* gene	
Other types of hypoparathyroidism

- Hypocalciuric hypercalcaemia syndrome type 2 is linked to mutations of the *GNA11* gene, located on chromosome 19p13.3 and coding for one of the sub-units of the G-protein (G-α11). This forms comprises about 10% of familial benign hypercalcaemia cases [23].
- Type 3 consists of mutations of the *AP2S1* gene, also located on chromosome 19 but at 19q13.3, generally at the level of arginine in position 15. *AP2S1* mutations account for about 20% of familial hypocalciuric hypercalcaemia cases. This form is associated with a more severe FHH variant that may lead to symptomatic hypercalcaemia with hypophosphoraemia and an increase of PTH with age, low bone mineral density and cognitive dysfunction.,. The AP-2 complex is a heterotetramer composed of α, β, μ and σ sub-units binding clathrin to the vesicle membranes. These vesicles intervene in the internalisation of the receptors coupled with the G-proteins (GPCRs). Mutations of the σ sub-unit of AP2 decrease the sensitivity of the cells to the extracellular calcium and reduce CaSR endocytosis [24, 25].

- About 20% of hypocalciuric hypercalcaemia syndrome cases are not related to the identified genes, suggesting that still unknown genes are probably involved [26].

Besides the mutations of the genes involved in types 2 and 3 FHH, FHH syndromes related to a mutation of the *CASR* gene must be differentiated from hyperparathyroidism with normal PTH [27].

In the hypocalciuric hypercalcaemia syndromes, the familial character of the hypercalcaemia and a calcium to creatinine clearance ratio below 0.01 support familial benign hypercalcaemia rather than hyperparathyroidism, despite a gray zone.

There are also other autosomal dominant genetic forms of familial hypercalcaemia, which are associated with different syndromic manifestations such as tumours and particularly hyperparathyroidism linked to tumour suppressor gene mutations, including:

- the *MEN1* gene, coding for menin in multiple endocrine neoplasia type 1 (MEN1),
- the *HRPT2 (/CDC73)* gene*,* specifically involved in carcinogenesis,
- the *CDKN1B* gene*,*
- but also the *APC, SFRPs, GSK3β, RASSF1A, HIC1, RIZ1,* and *WT1* genes, and possibly *CASR* [28], *GNA11*, *AP2S1, and GCM2* or glial cells Missing-2, a transcription factor linked to the early development pf parathyroid glands [1, 29]

as well as mutations of proto-oncogenes (*CCND1/PRAD1*, *RET* in MEN2, *ZFX*, *CTNNB1*, *EZH2*) [30–32].

Finally, mutations of the CYP24A1 gene with autosomal recessive transmission may induce a biological phenotype, characterized by hypercalcemia, hypercalciuria, depressed PTH, normal 25-OHD, increased 1-25-(OH)_2_D, and decreased 24-25-(OH)_2_D_3_ levels, characteristic of infantile hypercalcemia, which may also be related to *SLC34A1* gene mutations. Late-onset forms include nephro-lithiasis/-calcinosis, chronic renal insufficiency and hypertension. The *CYP24A1* gene belongs to the CYP450 group and encodes for 25-hydroxyvitamin D (25-OHD) 24-hydroxylase, a key enzyme of calcitriol (1-25-(OH)_2_D) degradation. Reduced rates of inactivation of 1-25-(OH)_2_D caused by a *CYP24A1* gene defect result in increased serum 1-25-(OH)_2_D levels and intestinal absorption of calcium, causing down-regulation of PTH secretion [33]. Two cases of hyperparathyroidism complicating CYP 24A1 mutations have been reported [34].

##### Treatment

Calcimimetics as a rule are not indicated except in case of symptomatic forms. It is important to remember to investigate for parathyroid adenoma in the event of complications.

#### Homozygous inactivating mutations

##### Clinical and laboratory presentation

The homozygous inactivating mutations of *CASR* [35, 36], which are much more rare, may result in severe neonatal hyperparathyroidism (NSHPT) with:

- marked hypercalcaemia,
- most of the time hypercalciuria, when very high PTH and calcium levels increase renal calcium excretion, with values of calcium/creatinine clearance ratio > 0.01
- increased PTH,
- dehydration,
- bone demineralisation,
- fractures.

Neonatal hyperparathyroidism is a severe life-threatening disorder. The role of normal maternal serum calcium levels perceived as being low by the newborn could promote the occurrence of this hyperparathyroidism, by stimulating PTH secretion, early during the intra-uterine life. The increased level of serum calcium leads to hypercalciuria.

Otherwise NSHPT is associated with a large proportion of *CASR* mutations involving the Ca^2+ -^binding sites particularly at the VFTD cleft, which is the principal site of Ca^2+^ binding [37]. Mutant sensors in the plasma membrane are major contributors to hyperplasia, which is usually observed in the four glands in NSHPT [17]. The main mutations reported in this condition are compound homozygous or heterozygous inactivating mutations of *CASR* or sometimes simply *de novo* occurring heterozygotes [37, 38], such as the R185Q and R227Q mutations, which are the cause of alterations in the MAP-kinase (MAPK) pathway.

##### Progression

Progression generally follows the familial benign hypercalcaemia pattern. Variants of this severe hypercalcaemia have been described with a later appearance in childhood, particularly in the case of compound heterozygosity of the *CASR* gene inherited from the parents each having asymptomatic familial benign hypercalcaemia, whereas inbred parents rather lead to homozygote children.

Finally, significant negative feedback from the protein coded by the *CASR* gene has ben reported in a considerable proportion of parathyroid carcinoma cases having a high proliferation index. In contrast, no mutation of the *CASR* gene has been demonstrated in these parathyroid carcinomas.

##### Treatment

Besides rehydratation with normal saline, treatment is based on cinacalcet (which is not always effective), biphosphonates, low-calcium milk and, as a last resort, on total parathyroidectomy [39].

### Hypocalcaemia via activating mutations of *CASR*

#### Hypercalciuric hypocalcaemia syndrome type 1

Hypercalciuric hypocalcaemia syndrome is an isolated form of autosomal dominant, congenital hypoparathyroidism that is the mirror of the presentation of FHH (Table 1). There is therefore hypocalcemia with normal or low PTH but that is maladjusted in all cases. The urine calcium is usually normal, consistent with hypercalciuria relative to the serum calcium levels. There is a tendancy for hypomagnesemia. This syndrome is linked to gain-of-function, or activating, mutations of the *CASR* gene [40–42].

- 50% of patients present with moderate and asymptomatic hypocalcemia that is found fortuitously,
- 50% present with paresthesias, tetany, epilepsy, severe hypocalcaemia, sometimes with Bartter syndrome [43],
- 10% present with hypercalciuria with nephrocalcinosis or lithiasis,
- Over 35% present with ectopic and/or basal ganglia calcifications.

The A843E, C131W, F788C mutations are generally associated with hypomagnesemia with PTH at the lower limit of normal. The P221L, K47N and finally E481K mutations are associated with normal serum magnesium levels, and an increased PTH in response to the hypocalcemia [44, 45].

#### Differential diagnosis

The differential diagnosis of these hypercalciuric hypocalcaemia syndromes consists of the hypercalciuric hypocalcaemia syndrome type 2 linked to a gain-in-function activating mutation of the *GNA11* gene (Table 2). The phenotype is identical to the hypercalciuric hypocalcaemia syndrome type 1 linked to an activating mutation of the *CASR* gene, apart from the hypercalciuria and hypomagnesemia that are not present in the type 2 form, [41, 46]. A gain-of-function mutation has still not been described for the *AP2S1* gene at this time.

The other differential diagnoses consist of hypoparathyroidism aetiologies.

This presentation may be reproduced by the presence of anti-CaSR stimulant antibodies, for which an investigation will therefore need to be done.

#### Clinical forms

##### Bartter-like phenotype

A Bartter-like phenotype of these *CASR* activating mutations results in a presentation of hypokalemic metabolic alkalosis, with moderate secondary hyperaldosteronism without very severe salt loss, but with a tendancy for hypercalciuric hypocalcemia. This presentation has been described in several adults and several children [43, 44].

It consists of mutations that are generally highly activating of the *CASR* gene, e.g. L125P.

The pathophysiology is related to CaSR activation, which inhibits the reuptake of sodium chloride by means of the thiazide-sensitive sodium chloride transporter. This effect is not visible when the mutation is only slightly activating, but becomes so when it is significant.

In this situation, the mutations that are usually responsible for Bartter syndrome (*NKCC2, ROMK, CLCKB* et *BSND*) are negative.

##### Sporadic hypoparathyroidism

There are cases of sporadic hypoparathyroidism that are identical to the autosomal dominant hypercalciuric hypocalcemia syndrome, except that the familial influence is lacking. These are therefore *de novo CASR* mutations, and must be differentiated from an autoimmune cause. The hypocalcemia may have few symptoms or may otherwise be very debilitating or even life-threatening. Few cases have been described.

#### Treatments

The emergency treatment for these types of hypocalcaemia is based on parenteral calcium administration and the standard of care treatment would be vitamin and calcium supplementation. Vitamin D stimulates the expression of *CASR* but causes an increase in urine calcium with the risk of nephrocalcinosis in 57% of treated subjects.

For this reason, it is recommended that treatment should only be given to patients with hypocalcaemia below 76 mg/L and/or who are symptomatic, using the smallest dose of 1 alpha-hydroxycholecalciferol (1 to 2 μg/day), while monitoring the 24-h urine calcium every 3 to 6 months. The combination with thiazides, to decrease the urine calcium, worsens the hypokalemic tendency [26]. Recombinant PTH may normalise the serum and urine calcium levels. Calcilytic compounds (allosteric inhibitors of CaSR) could be beneficial in the future, through stimulation of PTH secretion and reduction of urine calcium and renal calcifications [47, 48]. They are however usually ineffective in osteoporosis. Otherwise, certain drugs such as proton pump inhibitors may worsen hypomagnesemia and hypocalcemia and should be used with caution to avoid cardiac arrhythmias and seizures.

## Acquired causes

### Autoimmune hypocalciuric hypercalcaemia

The clinical presentation of autoimmune hypocalciuric hypercalcaemia is identical to the presentation of the genetically determined forms, with moderate hyperparathyroidism, relative hypocalciuria, and inappropriate PTH. An association with other autoimmune diseases is possible in the case of autoimmune polyendocrinopathy syndrome type 1 (APECED) or type 2 (particularly thyroid diseases and coeliac disease). The case studied histologically did not show lymphocytic infiltration of the parathyroids. Anti-CaSR antibodies, blocking in vitro*,* directed against the extracellular portion of the protein have been identified [49–51]. They inhibit the activation of CaSR by the extracellular calcium, resulting in PTH stimulation.

### Acquired autoimmune hypoparathyroidism

These types of hypoparathyroidism may occur in isolation or in association with autoimmune polyendocrinopathy type 1 or more rarely with type 2. They are characterised by the presence of antibodies directed against the extracellular portion of CaSR. The prevalence of these antibodies in isolated autoimmune hypoparathyroidism is about 49%. The presence of this type of antibody does not imply that they are necessarily functional.

Anti-*NALP5* antibodies have also been identified, particularly in cases of hypoparathyroidism related to the APECED syndrome. Their specificity is 50% and their sensitivity is 26%, which makes them a poorer indicator than the anti-CaSR antibodies,which have a specificity of 83% and a sensitivity of 50%. The presence of these antibodies in this type of syndrome is more frequent when they are assayed early relative to the date of occurrence of the hypoparathyroidism [50].

## Calcium-sensing receptor and other diseases

### Hypercalciuria and lithiasis

The serum calcium level is genetically determined for 50 to 70% of its variability. The A986S variant, as well as the R990G and Q1011E variants, are associated with higher serum calcium levels [27, 28] than in the general population. Hypercalciuria is associated with the R990G variant of *CASR* [18–20]. The minor allele rs6776158 predisposes to kidney stones by decreasing the transcriptional activity of the CASR gene promoter 1 and its renal tubular expression. Moreover, Claudin 14, a protein which regulates the transport of ions ans solutes at epithelial tight junctions, is expressed at a lower level in the rs6776158 GG homozygous subjects. In these cases, nephrolithiasis may occur by another mechanism than hypercalciuria [1, 19].

### Calcium-sensing receptor and the digestive tract

Hypercalcaemia is associated with increased acid discharge, since the secretion of gastrin and calcitonin is stimulated by the hypercalcaemia via the intermediary of the CaSR present in the gastrin-secreting cells. Patients presenting with hypercalcaemia therefore have a frequent tendency for hypergastrinaemia, which may explain the increased frequency of digestive disorders. CaSR reportedly has anti-inflammatory, anti-secretory, pro-absorbent and inhibitory properties on intestinal motility. Its activation could provide a new therapeutic approach for diarrhoea [52, 53].

### Calcium-sensing receptor and the respiratory tract

The activation of CaSR also reportedly has anti-inflammatory and anti-allergic properties, which could provide a therapeutic benefit [54].

### Calcium-sensing receptor and cancer

A correlation was observed between certain *CASR* rs17251221 type polymorphisms and coronary artery disease, type 2 diabetes, cancer and mortality. In cancer, CaSR appears to have paradoxical roles, and depending on the tissue involved, it is able to prevent or promote tumour growth. This effect would be mediated through genetic or epigenetic mechanisms such as methylation of the *CASR P2* promoter especially in colon cancer and neuroblastic tumors [1]. In tissues like the parathyroid or colon, CaSR inhibits proliferation and induces terminal differentiation of the cells. Therefore, loss of the receptor, as seen in colorectal or parathyroid tumours, confers malignant potential, suggestive of a tumour suppressor role. In contrast, in prostate and breast tumours, expression of CaSR is increased, and it seems that it favours metastasis to the bone, acting as an oncogene [55, 56]. Normal and neoplastic mammary epithelial cells express CaSR. During lactation, CaSR activation in the mammary cells causes negative feedback of parathyroid hormone-related protein (PTHrP) in the milk and blood and increases calcium transport in the milk. Conversely, in breast cancer, CaSR stimulates the expression of PTHrP. A switch in the function of the G-proteins underlies the opposite effects of CaSR on the expression of PTHrP in normal and neoplastic mammary epithelial cells.

### Calcium-sensing receptor and cardiovascular risk

CaSR is present on the β cells and the enteral endocrine cells, as well as on the adipocytes and the myocytes. This may explain why alterations in cardiac function and metabolic regulation are associated with genetically determined signalling abnormalities of CaSR, particularly with regard to insulin secretion, postprandial blood glucose regulation, lipolysis and inhibition of myocardial cell proliferation [57–63].

### Calcium-sensing receptor and taste regulation

CaSR could participate in taste control (particularly the taste of kokumi) and the regulation of enterogastric hormones, such as ghrelin, which are orexigenic and participate in glucose regulation in response to the amino acid intake in the intestine [64].

Knowledge of the calcium-sensing receptor enables the development of various approaches, especially those of an imaging agent (*Calhex-231*) in parathyroid diseases and perhaps in the medullary thyroid cancer.

## Calcium-sensing receptor modulators

There are two known types of CaSR modulators: CaSR agonists (or activators, still named calcimimetics) and calcilytic antagonists (or inhibitors of the calcium-sensing receptor) [48, 60, 65–67].

### CaSR agonists or calcimimetics

These drugs are capable of making CaSR more sensitive to serum calcium levels, thereby decreasing the parathyroid hormone and the serum calcium in hyperparathyroidism, whether they are primary, secondary or neoplastic. Two types have been described: type I, which is a direct agonist, and type II, which is a positive allosteric activator.

Cinacalcet is a type II calcimimetic and thus results in a risk of dose-dependent hypocalcaemia by reducing the parathyroid hormone. In patients on dialysis, cinacalcet reduces calcium, phosphorus and FGF23 levels, hyperplasia of the parathyroids and bone remodelling, with a bone gain of around 3% in the femur; it may diminish skeletal fracture rates and the need for parathyroidectomy. It is also indicated in primary hyperparathyroidism, parathyroid carcinomatosis, parathyromatosis, and refusal or contraindications of parathyroid surgery.

The second generation type II calcimimetic, Velcalcetide (*AMG416*) is currently under regulatory review.

Their benefits have also been discussed in patients with arterial hypertension and vascular calcifications. The *R-568* or *AMG641* calcimimetics are in fact capable of increasing CaSR expression and decreasing mineralisation of the vascular smooth muscle cells, which could have a beneficial effect on vascular calcifications [68]. Finally, the combination of cinacalcet with small doses of vitamin D has been shown to be effective in slowing down the progression of vascular calcifications compared with treatments using larger doses of vitamin D alone.

### Calcilytic antagonists or inhibitors of the calcium-sensing receptor

Calcilytics are CaSR antagonists that stimulate the secretion of PTH and reduce renal excretion of calcium. They have not been shown to be effective in osteoporosis, especially post-menopausal osteoprosis. They could be indicated in cases of idiopathic hypocalcaemia and hypercalciuria.

## Future outlook and Conclusions

There are many CaSR diseases, but familial hypocalciuric hypercalcaemia syndrome is the most common, even if it may sometimes be associated with, depending on the variant, a hypercalciuric tendency, thus posing problems for the differential diagnosis with hyperparathyroidism.

The genetic causes identified in adults usually require no treatment. Some cases of parathyroid adenomas, however, are associated with *CASR* gene mutations, thus justifying additional morphological investigations in case of severe hypercalcaemia, since excision of these adenomas can improve serum calcium levels.

The *CASR* gene might also be involved in tumourigenesis, particularly in the colon, breasts and the prostate, as well as in cardiovascular and inflammatory diseases, including both digestive and respiratory.

It is important to know how to make the diagnosis in complex situations, such as mammary neoplasm or chronic renal failure, since these disorders, which remain asymptomatic for a long time, can suggest neoplastic hypercalcaemia or that related to tertiary hyperparathyroidism, while it is in fact a genetically determined disorder.

## Abbreviations

*CASR*: Calcium-sensing receptor gene
CaSR: Calcium-sensing receptor: (protéine)
FHH: Familial hypocalciuric hypercalcaemia
GPCR: G-protein coupled receptor
PTH: Parathyroid hormone
